# Supplementary material for: Whole-brain mapping of spinal-projecting neurons in larval zebrafish
Source: eLife. 2026 Oct 29;15:RP112062. doi: 10.7554/eLife.112062 (PMC13719168; doi:10.7554/eLife.112062)
Supplement: Peer review history

# Editor report: eLife Assessment

**DOI:** 10.7554/eLife.112062.3.sa0

**Authors:** David Schoppik

**Affiliations:** NYU Grossman School of Medicine United States

**Keywords:** Compelling, Valuable

This **valuable** study is a rigorous and comprehensive anatomical description of populations of spinal-projecting neurons in the brain of the larval zebrafish. The **compelling** data reveal a far more complete and robust visualization of these populations than past work. The scholarship is also notable, featuring thoughtful contextualization of the findings with respect to both non-mammalian and mammalian literature.

# Referee report: Reviewer #2 (Public review)

**DOI:** 10.7554/eLife.112062.3.sa1

Summary:

Vertebrate spinal cord receives inputs from many supraspinal regions. The authors used optical backfilling to trace neurons in the zebrafish larval brain sending axons to the spinal cord . With two-photon microscopy, they managed to render a comprehensive 3D map of these neurons and drew homologs with mammalian brain structures.

Strengths:

The main strength lies in the precise 3D mapping. The fact that most of previously reported neuron groups have been confirmed by their approach is a solid endorsement of their methodology.

This study provides a comprehensive alternative anatomical reference framework for studying individual groups of supraspinal neurons with projections to the zebrafish spinal cord.

Weaknesses:

The backfilling approach does not reveal the full trajectories of axons, which is available to some degree by ZExplorer Atlas.

# Author comment: Author response

**DOI:** 10.7554/eLife.112062.3.sa2

**Authors:** Martin Carbo-Tano, Kevin Fidelin, Tom Welch, Sujatha Narayan, Misha B Ahrens, Réjean Dubuc, Claire Wyart

**Affiliations:** Institut du Cerveau Paris France; Institut du Cerveau Paris France; Institut du Cerveau Paris France; Janelia Research Campus Ashburn United States; Howard Hughes Medical Institute Ashburn United States; Université de Montréal Montréal Canada; Institut du Cerveau Paris France

The following is the authors’ response to the original reviews.

**Reviewer #1 (Public review):**

Summary:

The authors have achieved an excellent, thorough anatomical characterization of all spinal projecting neurons in the larval zebrafish. The comprehensive nature of their labeling approach and their quantification will make this work an instant reference benchmark for a wide number of zebrafish researchers. In addition, the scholarly approach in comparisons with other work in and outside of fish makes the manuscript valuable to researchers outside the field who would like to know how to translate between zebrafish and mouse terminologies.

Strengths:

The figures are clear and easy to follow. The literature review is impressive. The authors are careful to note the few limitations of their approach (eg the absence of Mauthner cell labeling and associated large neuron weak label). The manuscript does the whole field a major service.

Weaknesses:

No weaknesses were identified by this reviewer.

We deeply thank reviewer #1 for recognizing the importance of our work.

**Reviewer #2 (Public review):**

Summary:

The vertebrate spinal cord receives inputs from many supraspinal regions. The authors used optical backfilling to trace neurons in the zebrafish larval brain sending axons to the spinal cord. With two-photon microscopy, they managed to render a comprehensive 3D map of these neurons and drew homologs with mammalian brain structures.

Strengths:

The main strength lies in the precise 3D mapping. The fact that most of the previously reported neuron groups have been confirmed by their approach is a solid endorsement of their methodology. This study provides a comprehensive alternative anatomical reference framework for studying individual groups of supraspinal neurons with projections to the zebrafish spinal cord.

Weaknesses:

The whole approach could be enhanced by counter-staining their preparation to profile brain structures, including many nuclei more precisely.

Also, the backfilling approach does not reveal the full trajectories of axons, which is already available to some degree by ZExplorer Atlas.

We thank Reviewer #2 for the attention given to important details. As you will read below, our approach needed to be implemented on live larval zebrafish for the PA GFP to fluoresce only when photactivated. Although counterstains on fixed samples could have been done, it would have been difficult to trace it back in a fixed (and deformed) sample where all neurons appear GFP-positive. We wrote this publication so it can serve the field best before the release of full brain connectome in 2027. Further molecular and histological characterization will be the topic of a subsequent study. Below we will individually address the issues stated by the reviewer.

**Reviewer #2 (Recommendations for the authors):**

(1) The power of 800 nm laser: 1 mW in Figure 1a, 10 mW stated in the method text.

Thanks for catching this, this is now corrected to 1 mW in the revised manuscript.

(2) Figure 2c: laterality density (e.g., 90:40 by neuron numbers) is already quite clear. The laterality index is less informative if not causing confusion. Take POA as an example; shouldn't the value be close to 1, rather than -0.8 based on your formula?

The laterality density plot shows the anatomical spatial distribution of spinal descending neurons relative to the midline, whereas the laterality index is a scalar summary of the relative neuron counts on each side, independent of spatial position. For POA, we observed after pooling across animals 26 ipsilateral versus 1 contralateral neurons, which gives an LI close to -0.8 (after bootstrapping), indicating strong ipsilateral dominance. The density plot additionally shows that these neurons cluster close to the midline. We view the two measures as complementary: one quantifies laterality, the other reflects its spatial organization.

(3) Line 286, check grammar.

Corrected to "Using the ZExplorer atlas (Du et al., 2025), which contains thousands of single-neuron tracings in the larval zebrafish brain, we searched for telencephalic neurons projecting to the spinal cord."

(4) Add discussion on comparison between your approach, conventional backfill tracing method, singlecell tracing by dye or GFP, and, more importantly, ZExplorer Atlas.

We believe this comparison is sufficiently addressed in the Introduction and Discussion.

(5) Line 346, add "in bi" after "scale bar".

Corrected, thank you for spotting this.

(6) Figure 4, change label ICN to INC in c and d.

We apologize for this confusion, all of them are now corrected in the revised manuscript.

(7) Line 389, change to "ventral (top) to dorsal (bottom)". Try to use the same order for all figures, i.e., dorsal images on top and ventral ones at the bottom.

Corrected for the specific line and now all figures follow the same convention.

(8) Figure 7b, add stack location diagrams.

Added. Thank you very much for suggesting an improvement to the figure readability.

(9) Figure 7c and other figure panels, suggest changing the word "medial" to "middle" since medial/lateral is usually used to describe sagittal locations.

Good point: now corrected as suggested.

(10) Why was DAPI counter-staining not carried out? This would help the authors to better draw outlines for different nuclei. It also provides important data on the proportion of SPNs within each nucleus.

We agree with the reviewer that determining the proportion of SPNs within each nucleus is an important question; however, properly addressing this requires an independent, cytoarchitecture-based definition of nucleus boundaries, a substantial project that we consider outside the scope of the present study.

DAPI labels cell nuclei generally but carries limited information relevant to classical neuroanatomical delineation of nuclei. Methods such as Nissl staining are better suited to this purpose, as they reveal the cytoarchitectonic characteristics of neurons. As mentioned in the Introduction and Discussion, we instead based our nuclei definitions on the strong evolutionary conservation of spinal-projecting neuron distributions, which we used as a proxy for delineating the different nuclei.

(11) The photoactivation is restricted to the rostral spinal cord. Therefore, how far the axons project throughout the spinal cord cannot be assessed with the current photo-activation method.

We agree with the reviewer: because photoactivation was restricted to the rostral spinal cord, our current data cannot resolve how far individual axons project caudally. However, all evolutionary comparisons in this study were performed across vertebrates using similarly rostrally-labeled SPNs, ensuring consistency in our cross-species analysis. We therefore do not believe that this limitation undermines our initial characterization of the SPN populations.

We agree, however, that extending photoactivation to multiple spinal levels will be valuable for future work characterizing and refining the nuclei proposed in this work. Some of this effort is carried for vsx2+ neurons only by Jia et al., under revision in Nature Communications.

(12) Line 544, if it is the spinal trigeminal tract, why does there appear to be some feint soma staining?

We apologize for the confusion. We always meant to say trigeminal nucleus and not its tract. Text and figure legend are now corrected.

**Reviewer #3 (Public review):**

In this study, the authors aim to provide the most comprehensive and detailed topographic map to date of spinal projection neurons in the larval zebrafish brain. They achieve this by retrogradely photoactivating, in the rostral spinal cord, a photoconvertible GFP expressed pan-neuronally, and by constructing a template larval zebrafish brain atlas to regionalize the location of all labeled somata across the brain. The labeling strategy, together with the chosen animal model, provides strong support for the completeness of the dataset. The generation of a standardized anatomical atlas establishes a rigorous framework for analysis. Molecular and anatomical evidence suggesting evolutionary conservation of selected regions of interest appears solid.

Overall, the authors successfully achieve their aim. By generating this atlas of spinal projection neurons, they provide not only an anatomical framework of the regions involved, but also an important reference for improving the orientation and regionalization of the zebrafish brain, which has historically been difficult to define. This work may serve as a valuable resource for future evolutionary, developmental, and comparative studies of spinally projecting neuronal populations implicated in diverse functions.

We deeply thank Reviewer #3 for recognizing the impact of our work and benefit for the community at large, which has always been our intention.
